# Does lifting female piglets by one hind leg increase the risk of umbilical and hind leg lesions?

**DOI:** 10.1186/s40813-024-00413-6

**Published:** 2024-12-20

**Authors:** Kristiane Barington, Marie Høy Hansen, Amanda Bastian Andersen, Ken Steen Pedersen, Inge Larsen

**Affiliations:** Faculty of Health and Medical Sciences, Department of Veterinary and Animal Sciences, University of Copenhagen, Ridebanevej 3, Frederiksberg C, 1870 Denmark

**Keywords:** Abdominal wall closure, Omphalitis, Porcine, Umbilical hernia, Umbilical outpouching

## Abstract

**Background:**

Umbilical lesions in pigs have a negative impact on animal welfare and productivity. It has been suggested that lifting young piglets by one hind leg may be a risk factor for developing omphalitis and umbilical hernia. However, the hypothesis that lifting piglets by one hind leg should stretch the umbilical wall and impede the healing of the umbilicus has not yet been investigated. The present study examined if piglets caught, lifted, and carried by one hind leg have an increased risk of developing lesions in the umbilicus and the hind legs compared to piglets caught, lifted, and carried with support under the abdomen.

**Materials and methods:**

In a commercial indoor sow herd, 1901 piglets were randomly allocated into two groups on the day of birth. Piglets in Group 1 (986 piglets) were caught, lifted, and carried by one hind leg (either left or right, as the same leg was not necessarily used each time). Piglets in Group 2 (915 piglets), were caught, lifted, and carried with support under the abdomen. All piglets were lifted 8–10 times during the first 14 days of life as a part of routine management procedures. From each group, 50 female piglets, 14 days old, were randomly selected and euthanised for necropsy and histopathological evaluation.

**Results:**

The risk of having haemosiderophages in the umbilicus was 1.4 times higher in piglets caught, lifted, and carried by one hind leg compared to piglets caught, lifted, and carried with support under the abdomen (*p* = 0.01). No other variable differed significantly between the groups. Omphalitis was present in 68% and 58% of piglets in Groups 1 and 2, respectively. Moreover, umbilical herniation was present in 14% and 12% of piglets in Groups 1 and 2, respectively. Lesions were present in the hind legs of piglets in both groups and included synovial hyperplasia, neutrophilic granulocyte infiltration, oedema, and haemorrhage.

**Conclusion:**

Female piglets caught, lifted, and carried by one hind leg did not have an increased risk of umbilical hernia, omphalitis, or joint lesions compared to piglets caught, lifted, and carried with support under the abdomen.

## Background

Newborn piglets are at risk of developing lesions related to the umbilicus. The rupture of the umbilical cord causes a wound through which pathogens may enter and cause infectious omphalitis and subsequently increase the risk of developing septicaemia, arthritis, meningitis, peritonitis, and umbilical herniation [1–4]. Umbilical hernias and differential diagnoses causing an umbilical outpouching (UO) are conditions often encountered in pigs [5–7]. UOs negatively affect productivity causing e.g. decreased growth rate, increased mortality rate due to euthanasia, and early slaughter [5, 7, 8]. Moreover, UOs may compromise animal welfare as pigs with UOs exhibit abnormal behaviour, and may suffer from skin ulcerations, intestinal obstruction, infarction, and incarceration [9–11]. Intestinal lesions like intestinal obstruction, infarction, and incarceration are known to cause pain in humans, and it is reasonable to assume that it applies to other species as well [12]. In Denmark, UOs in pigs are common and Hansen et al. 2024 estimated that nearly 1 million pigs out of a production of 32 million pigs are affected yearly [5]. In that study, 30 Danish conventional herds were included and the average prevalences of piglets and weaners presenting UO were 4.2% and 2.9%, respectively [5]. Searcy-Bernal et al., 1994 reported a cumulative incidence rate of umbilical hernia of 1.5% in a commercial swine herd in California.

The average umbilical cord length of a porcine foetus at term ranges between 17 and 50 cm [13]. However, during parturition the umbilical cord breaks due to the strain put on the cord when the piglet is expulsed. Excessive stretching of the umbilical cord during farrowing has been suggested as a risk factor for developing umbilical hernia [14]. Additionally, excessive stretching causes premature rupture of the umbilical cord and thereby asphyxia, potentially leading to decreased growth rates, higher mortality, and stillbirths [13, 15]. Recent studies report that the prevalence of piglets born with an intact umbilical cord ranges between 79% and 86% [16–18].

It has been proposed that grasping and lifting piglets by one hind leg causes a stretch on the abdominal wall that hampers the healing of the umbilical area and increases the risk of lesions at the umbilicus [5, 19]. Lifting piglets by one or both hind legs is an accepted handling practice [20, 21]. In conventional pig production piglets are managed several times during the first days of their lives e.g., clinical examinations, transfers between sows, treatments, vaccinations, castration, tail docking, and insertion of ear tags. However, it has not yet been investigated to what extent the lifting technique affects the healing of the umbilical area. Female pigs have an increased risk of developing UOs compared to males [5, 10]. Hansen et al., 2024 reported a prevalence of UO of 5.7% in female piglets and 3.8% in male piglets [5]. Similarly, Hansen et al., 2021 found a prevalence of UO of 11.6% in female pigs and 7.9% in male pigs [10]. Therefore, the present study aims to investigate if more supportive handling of female piglets during the first two weeks of life can reduce gross and histological lesions in the abdominal wall at the umbilicus. Additionally, to evaluate if the lifting technique impacts gross and histological lesions in the hind legs of female piglets.

## Materials and methods

### Study design

The study included all live-born piglets from eight farrowing batches that were born on Mondays and Tuesdays from August to November 2023. The litters were randomly allocated into two groups on the day of birth. Piglets in Group 1 were caught, lifted, and carried by one hind leg (either left or right, as the same leg was not necessarily used each time). Piglets in Group 2, were caught, lifted, and carried with support under the abdomen. All piglets were lifted 8–10 times during the first 14 days of life as part of routine management procedures e.g., shortening of the umbilical cord to a length of 2–5 cm, clinical examinations, toltrazuril and iron treatments, tail docking, litter exchange, and insertion of ear tags. The sow number was recorded at birth and again on day 14 to monitor whether the frequency of moving piglets, was similar between the groups.

From each group, 50 female piglets, 14 days old, were randomly selected and euthanised. The piglets were anesthetized by an intramuscular injection of zolazepam and tiletamine (Zoletil^®^Vet., 50 mg/mL, Virbac Danmark A/S, Kolding, Denmark) until loss of the corneal reflex. Then they were euthanised by intracardial injection of pentobarbital sodium (Euthasol^®^, 400 mg/mL, Dechra Veterinary Products A/S, Uldum, Denmark). All euthanised piglets were transported to the University of Copenhagen (1.5 h drive), stored at 4 ºC, and subsequently necropsied within 24 h of euthanasia.

This study originates from a clinical field study of pigs followed from birth to 9–10 weeks of age (unpublished). The sample size for the clinical field study was determined based on a clinical assessment of the prevalence of UOs with an expected reduction in UO prevalence from 5 to 2.5%. For a two-sided test with a significance level of 0.95 and power of 0.8, a sample of 2000 pigs was required for the clinical field study. Gross and histopathological examination is regarded as the gold standard for diagnosing umbilical lesions. Therefore, in the present study, a total of 100 euthanised piglets were considered sufficient to detect potential differences between the groups.

### Herd description

The study was carried out in a Danish commercial indoor sow herd consisting of 880 sows. Sows were housed in farrowing crates (2.0 m x 2.9 m) with a solid concrete floor beneath them and a slatted cast-iron floor in the rear of the pen. The piglet nest was located in one corner of the pen, near the sow’s head, with a full concrete, heated floor, and heat lamps provided during the first week of the piglets’ lives. The room temperature was set to 20˚C. and the heat lamp was placed 50 cm from the floor. Piglets had ad libitum access to drinking water through small water nipples. As far as possible the herd cleansed and disinfected farrowing pens with Virkon^TM^S (Potassium monopersulfate, sulfamic acid, detergents, active oxygen, auxiliary agents, and red indicator dye) at compartment/batch level. However, some 1-week-old piglets and their sows were housed in the compartments with sows in the process of farrowing.

Farrowings were monitored by the herd personnel during the daytime, and assistance was provided if the sow was straining without birth of a piglet and/or the last piglet born had a dry body surface. In cases requiring farrowing assistance, the herd personnel conducted a vaginal examination using disposable gloves covered in lubricant gel to manually extract the piglet. The herd prevalence of postpartum dysgalactiae syndrome (PDS) in the sows was 25–30%. Sows suffering from PDS were treated with benzylpenicillinprocaine, meloxicam, and oxytocin postpartum. Oxytocin was never used during farrowing.

In this herd routine procedures included shortening the umbilical cord to a length of 2–5 cm on the day of birth and disinfection of the umbilicus with Blue Spray (Aeropak A/S, Hedensted, Denmark) containing 60–100% ethanol. Antimicrobial treatment was carried out by the staff on indication, i.e. piglets that presented signs of bacterial infection were treated with either amoxicillin against lameness (arthritis) or a combination of sulfadoxin and trimethoprim against diarrhoea (intestinal infections). All piglets were tail docked.

### Necropsy

Piglets underwent a complete necropsy following the procedure outlined by Madsen and Jensen et al. 2011 [22]. Necropsies were conducted by two pathologists who were blinded to the group status of each pig. The depth, length, and width of the umbilicus were measured. All gross lesions including umbilical hernia were registered as present or absent. An umbilical hernia was defined as an unsuccessful closure of the abdominal wall at the umbilical ring through which the peritoneum protruded to form a hernial sac covered by skin [23]. The umbilicus and the synovial membranes from the tarsal joints were sampled for histopathological examination.

### Histopathology

**Fig. 1:**
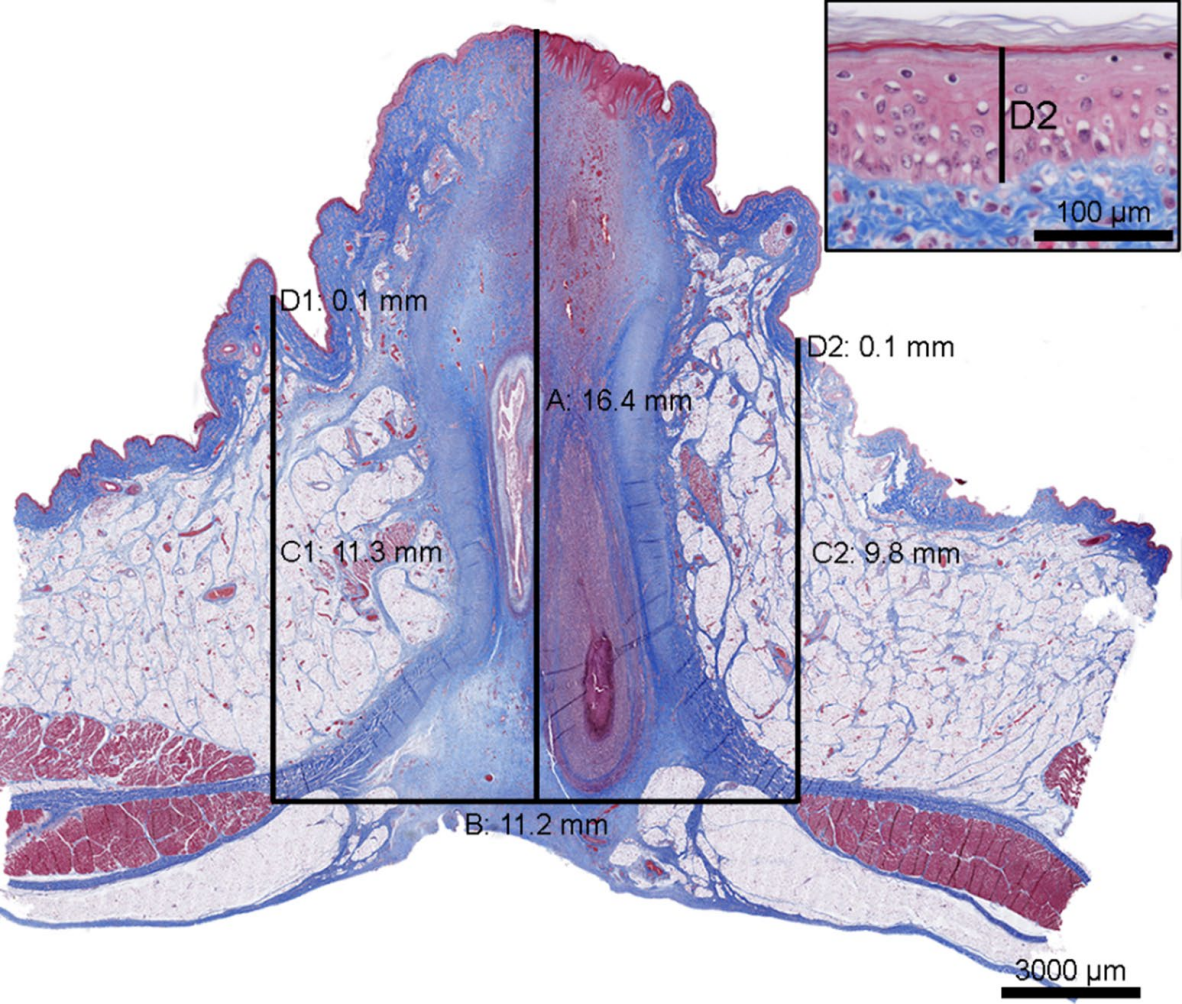
Tissue section of the umbilicus from a 14 day old piglet lifted by one hind leg (Group 1). The umbilicus is cut through the centre in the transverse plane, ventrodorsal direction. The thickness of the abdominal wall at the centre (**A**), the distance between the abdominal muscles (**B**), the thickness of the abdominal wall at the periphery of the umbilicus (C1 and C2), and the thickness of the epidermis (D1 and D2, insert) were measured. For the measurements C1 and C2 and D1 and D2 the average value was calculated for each. Masson’s Trichrome, scale bar: 3000 μm. Insert: Magnification of D2, scale bar: 100 μm

Tissue samples were fixed in 10% neutral buffered formalin for five to seven days. Following fixation, the umbilical tissue was trimmed by sectioning the tissue in the transverse plane in the ventrodorsal direction through the centre of the umbilicus (Fig. 1). The tissues were processed through graded concentrations of ethanol and xylene and embedded in paraffin. Tissue sections of 4–5 μm were stained with haematoxylin and eosin (HE) and Masson’s Trichrome [24, 25].

Tissue sections of the umbilici, stained with HE, were examined for lesions in the epidermis and the underlying connective tissue (Wharton’s jelly). Lesions were registered as present or absent. Omphalitis was defined as abscesses, granulomas, and/or diffuse infiltration of more than 40 neutrophilic granulocytes in a field of view of 0.238 mm^2^, in any area of the connective tissue. The cut-off value was based on a not yet published study in which less than 41 neutrophils in a field of view of 0.238 mm^2^ were found to be background inflammation due to the healing of the umbilicus and not infectious omphalitis [26].

**Fig. 2:**
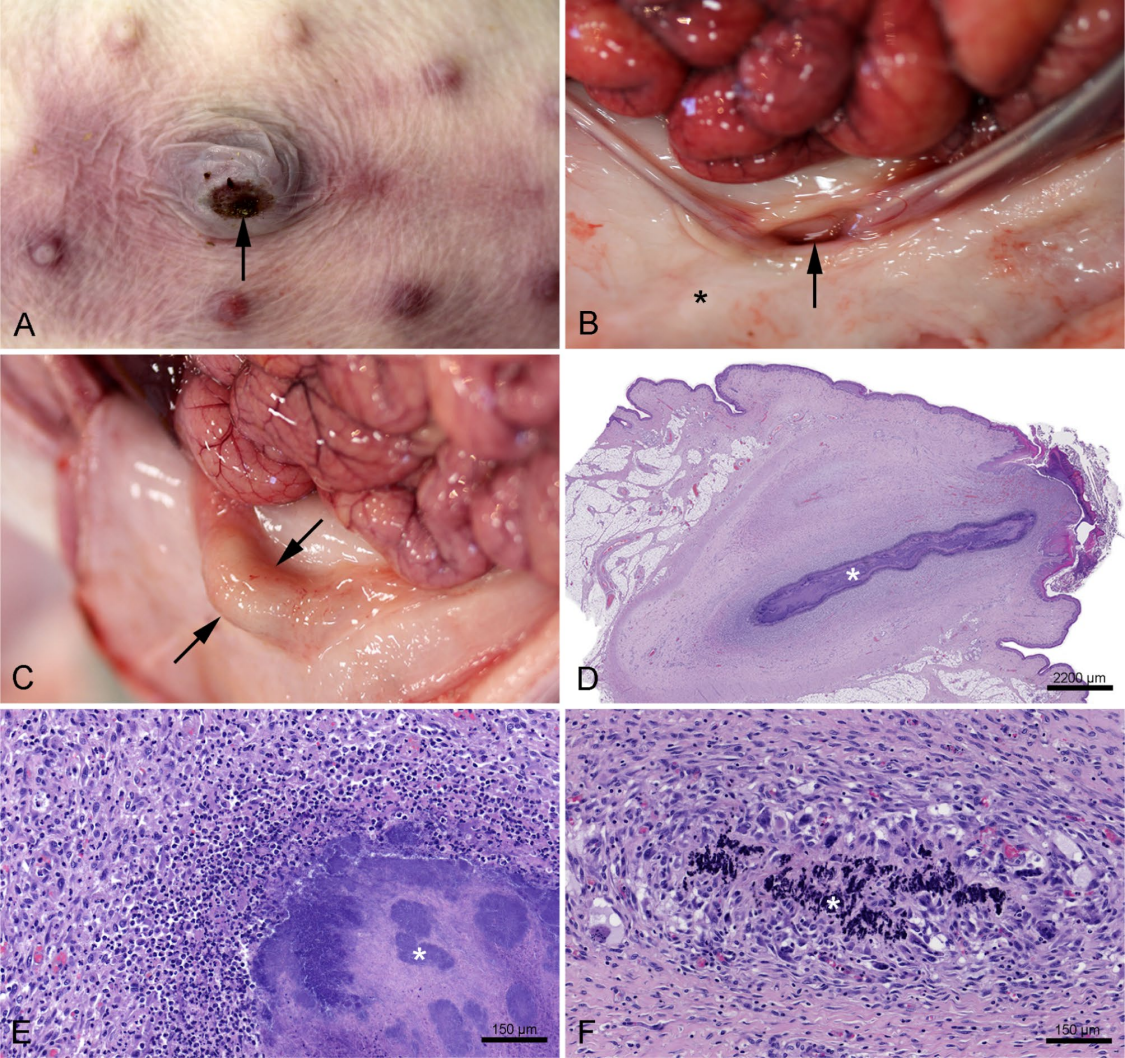
**A**: Umbilicus from a 14 day old piglet lifted with support under the abdomen (Group 2). The piglet presented with an umbilical hernia. The depth, width, and length of the hernia measured 12, 25, and 14 mm, respectively. A skin ulceration (arrow) measuring approximately 9 × 5 mm was present on the ventral surface of the hernia. **B**: Umbilical hernia from the piglet in Fig. 2A viewed from the inside of the abdomen. The closure of the abdominal wall (*) is incomplete leaving an umbilical ring (arrow) at the entrance to the hernial sac. **C**: Omphalitis in a 14 day old piglet lifted with support under the abdomen (Group 2). The lesion was characterized by an abscess (arrows) located at the umbilicus. No lesions were apparent from the skin surface i.e. before opening the abdominal cavity. **D**: Histological image of omphalitis in a 14 day old piglet lifted by one hind leg (Group 1). An abscess (*) is present within the umbilicus. Part of the lesion is shown in higher magnification in Fig. 2E. Haematoxylin and eosin, scale bar 2200 μm. **E**: Higher magnification of the umbilical abscess shown in Fig. 2D. The abscess consisted of a centre of bacterial colonies (*), surrounded by a massive infiltration of neutrophilic granulocytes and granulation tissue. Haematoxylin and eosin, scale bar 150 μm. **F**: Histological image of omphalitis in a 14 day old piglet lifted by one hind leg (Group 1). A granuloma is present in the connective tissue of the umbilicus. The lesion is characterized by a centre of mineralization (*) surrounded by multinucleated giant cells, epithelioid cells, macrophages, and proliferating fibroblasts. Haematoxylin and eosin, scale bar 150 μm

Tissue sections of the umbilici stained with Masson’s Trichrome were scanned using the Axioscan 7 (Zeiss, Germany) and measured in QuPath v0.5.1 [27]. The distance between the abdominal muscles, the thickness of the epidermis and the abdominal wall at the centre, and the periphery of the umbilicus were measured as shown in Fig. 1.

HE stained tissue sections of the synovial membranes were evaluated for lesions. All histological evaluations were conducted blinded to the group status of each pig.

### Statistics

The prevalences of piglets presenting gross and histological manifestations were calculated for Groups 1 and 2, respectively. For each categorical variable, the comparison of proportions between piglets in Group 1 and Group 2 was performed using Fisher’s exact test. Relative risks (RR) and associated 95% confidence intervals (CI) were calculated for gross and histological manifestations for piglets in Group 1 compared to piglets in Group 2. Limits of 95% CI not including 1 and a P-value below 0.05 were set as the criteria for statistical significance.

The average thicknesses of the epidermis and the abdominal wall at the centre and the periphery of the umbilicus were calculated for piglets in Groups 1 and 2. In addition, the average distance between the abdominal muscles was calculated for each group. Standard deviations (SD) were calculated for each variable. The data was checked for normal distribution on QQ plots, and an unpaired t-test was performed to identify significant differences between the groups. A P-value below 0.05 was set as the criteria for statistical significance.

In each group, piglets were subdivided into three categories i.e. (I) piglets with an umbilical hernia, (II) piglets with omphalitis, and (III) piglets with no umbilical lesions. Piglets presenting both an umbilical hernia and omphalitis were placed in category no. I (umbilical hernia). The average depth, length, and width of the umbilici were calculated for each category in each of the two groups. The SD was calculated for each variable. The data was checked for normal distribution on QQ plots, and an unpaired t-test was performed to identify significant differences between Group 1 and Group 2. A P-value below 0.05 was set as the criteria for statistical significance.

All calculations and analyses were executed in Microsoft Excel (Microsoft 365, Microsoft Corporation, Washington, USA) or GraphPad Prism version 10.2.3 (403) (GraphPad Software, Massachusetts, USA).

## Results

In total, 103 litters (*n* = 1901 piglets in total) were allocated into Group 1 (*n* = 52 litters, 986 piglets in total) or Group 2 (*n* = 51 litters, 915 piglets in total). From each group, 50 female piglets were subjected to necropsy and histopathological evaluation. The average body weights of the piglets at necropsy were x̄ = 3.6 ± 0.9 kg and x̄ = 3.5 ± 0.9 kg in Groups 1 and 2, respectively. In Group 1, 10 out of 50 piglets were treated with antimicrobials due to diarrhoea (*n* = 4 piglets), lameness (*n* = 1 piglet), and symptoms not registered (*n* = 5 piglets). Similarly in Group 2, 8 out of 50 piglets were treated with antimicrobials due to diarrhoea (*n* = 4 piglets), lameness (*n* = 1 piglets), and symptoms not registered (*n* = 3 piglets).

At 14 days of age, 42 out of 50 piglets in Group 1 and 44 out of 50 piglets in Group 2 were found with a different sow than at birth, indicating a similar frequency of movement between the two groups (*P* = 0.774).

### Umbilicus

Pathological manifestations (gross and histological) at the umbilicus included umbilical hernia, skin ulceration, hyperkeratosis, rete peg formation, haemosiderophages, haemorrhage, and omphalitis (Table 1; Fig. 2A-F). The average size ± standard deviation of the umbilical ulcerations was x̄ = 5.9 ± 6.2 mm^2^ and x̄ = 8.9 ± 11.1 mm^2^ in Groups 1 and 2, respectively. Piglets with omphalitis presented abscess formation, granulomas, and/or diffuse infiltration of neutrophilic granulocytes in Wharton’s jelly (Fig. 2D-F). In piglets that received antimicrobial treatment, omphalitis was diagnosed in 6 out of 10 piglets (60%) in Group 1 and in 8 out of 8 piglets (100%) in Group 2. Among non-treated piglets, omphalitis was diagnosed in 28 out of 40 piglets (70%) in Group 1 and in 21 out of 42 piglets (50%) in Group 2.

Wharton’s jelly consisted of either mature fibrous tissue or a mixture of granulation tissue and mature fibrous tissue (Table 1; Fig. 2D). The prevalence of piglets with haemosiderophages in Wharton’s jelly differed significantly between the two groups (Table 1).

**Fig. 3:**
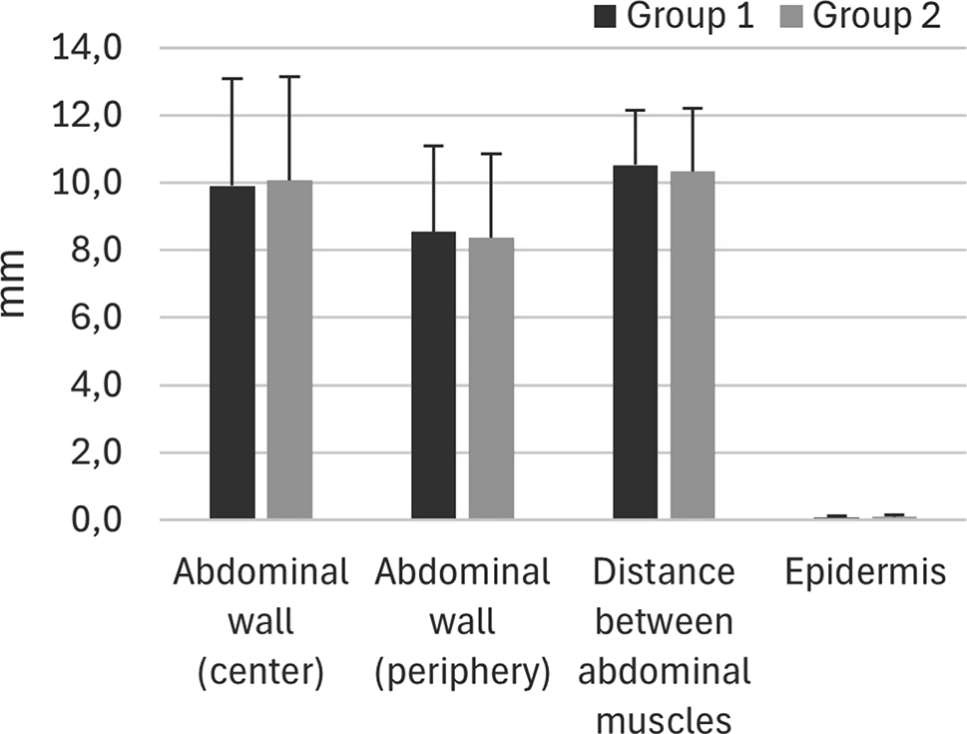
The average thicknesses (mm) of the epidermis and the abdominal wall at the centre and periphery of the umbilicus in piglets from Group 1 (dark grey) and Group 2 (light grey). Moreover, the average distance (mm) between the abdominal muscles is depicted for each group. The error bars show the standard deviation. The measurements were conducted on Masson’s Trichrome stained tissue section of the umbilicus from 41 out of 50 piglets and 46 out of 50 piglets from Groups 1 and 2, respectively. There were no significant differences between the groups, *P* > 0.05

The dimensions of the abdominal wall were measured on Masson’s Trichrome stained tissue sections of the umbilicus from 41 to 46 piglets from Groups 1 and 2, respectively. The remaining tissue sections (*n* = 9 and *n* = 4 in Groups 1 and 2, respectively) were excluded due to sampling and preparation errors i.e., lack of abdominal muscle tissue and/or loss of epidermis. The thicknesses of the abdominal wall at the centre and the periphery of the umbilicus, the thickness of the epidermis, and the distance between the abdominal muscles did not differ significantly between the two groups (Fig. 3).

**Fig. 4:**
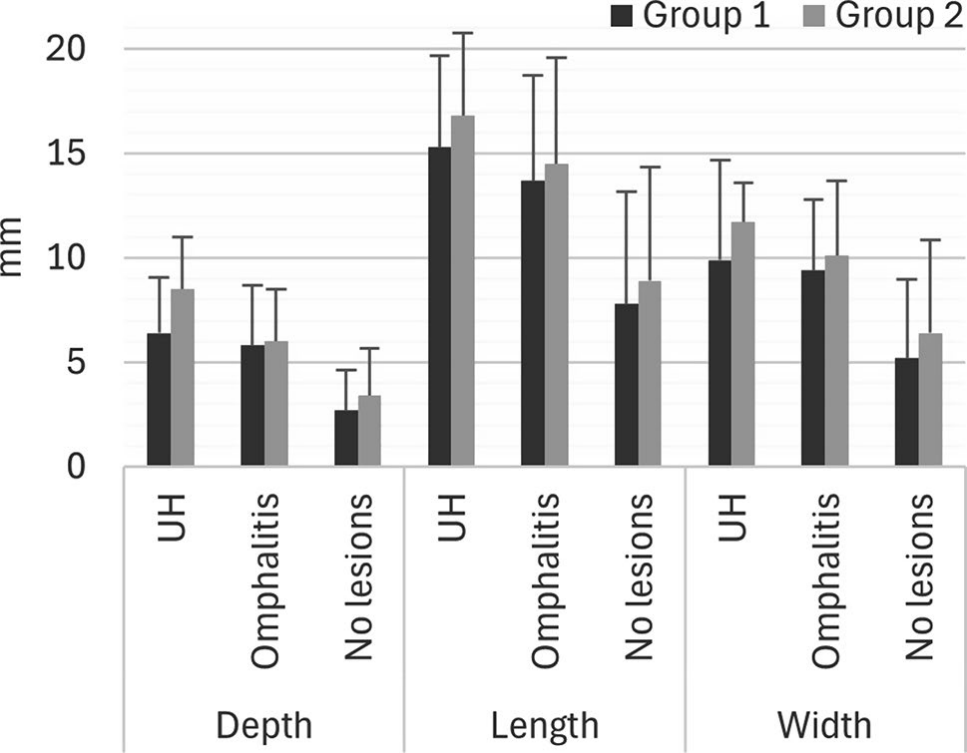
The average depth, length, and width (mm) of the umbilicus of piglets with umbilical hernia (UH), omphalitis, or no umbilical lesions in Group 1 (dark grey, *n* = 50 piglets) and Group 2 (light grey, *n* = 50 piglets). The measurements were performed on a total of 100 dead piglets before necropsy. The error bars show the standard deviation. There were no significant differences between Group 1 and Group 2, *P* > 0.05

The gross dimensions of the umbilicus of piglets presenting an umbilical hernia, omphalitis, or without lesions, are presented in Fig. 4. 5 out of 13 piglets with umbilical hernia also presented omphalitis.

**Table 1:** Prevalence of female piglets with gross and histological manifestations in the umbilicus in Group 1 (piglets caught, lifted, and carried by one hind leg) and Group 2 (piglets caught, lifted, and carried with support under the abdomen), respectively. In addition, the P-value, the relative risk (RR), and the associated 95% confidence interval (CI) of each variable for piglets in Group 1 compared to piglets in Group 2 are presented. WJ = Wharton’s Jelly. *Statistically significant

Manifestations	Group 1 *n* = 50	Group 2 *n* = 50	RR (95% CI)	*P*-value
*Gross manifestations*				
Umbilical hernia	14%	12%	1.2 (0.4–3.1)	> 0.999
Ulceration	42%	52%	0.8 (0.5–1.2)	0.423
*Histopathological manifestations*				
Hyperkeratosis	100%	100%	-	-
Rete peg formation	96%	98%	1.0 (0.9–1.1)	> 0.999
Omphalitis	68%	58%	1.2 (0.9–1.6)	0.408
Abscess	46%	44%	1.1 (0.7–1.6)	> 0.999
Granuloma	8%	8%	-	-
Diffuse inflammation	60%	56%	1.1 (0.8–1.5)	0.840
Bacteria	40%	44%	0.9 (0.6–1.4)	0.840
Haemosiderophages in WJ	86%	62%	1.4 (1.1–1.8)	0.011*
Haemorrhage in WJ	12%	12%	-	-
Granulation tissue in WJ	78%	88%	0.9 (0.7–1.1)	0.287

### Hind legs

The prevalence of piglets presenting gross changes in the joints of the hind legs did not vary between the groups (Table 2). The histological findings in the synovial membranes of the tarsal joints included infiltration of neutrophilic granulocytes and haemosiderophages, proliferation of synoviocytes, hyperaemia, haemorrhage, and oedema. The prevalences of these changes did not differ significantly between the two groups (Table 3).

**Table 2:** Prevalence of female piglets with gross manifestations in the hip, knee, and tarsal joints in Group 1 (piglets caught, lifted, and carried by one hind leg) and Group 2 (piglets caught, lifted, and carried with support under the abdomen), respectively. In addition, the P-value, the relative risk (RR), and the associated 95% confidence interval (CI) of each variable for piglets in Group 1 compared to piglets in Group 2 are presented

Gross manifestations	Group 1 *n* = 50	Group 2 *n* = 50	RR (95% CI)	*P*-value
Hyperaemic synovial membrane	42%	50%	0.8 (0.5–1.3)	0.548
Synovial proliferation	14%	26%	0.5 (0.2–1.2)	0.210
Increased synovial fluid	12%	12%	-	-
Haemarthron	4%	0%	-*	0.4949
Periarticular subcutaneous oedema	28%	20%	1.4 (0.7–2.8)	0.483

* The number of piglets presenting haemarthron in Group 2 is zero. Therefore, the relative risk becomes infinite

**Table 3:** Prevalence of female piglets with histological lesions in the tarsal joints in Group 1 (piglets caught, lifted, and carried by one hind leg) and Group 2 (piglets caught, lifted, and carried with support under the abdomen), respectively. In addition, the P-value, the relative risk (RR), and the associated 95% confidence interval (CI) of each variable for piglets in Group 1 compared to piglets in Group 2 are presented

Histological manifestations	Group 1 *n* = 50	Group 2 *n* = 50	RR (95% CI)	*P*-value
Synovial hyperplasia	32%	28%	1.1 (0.6–2.1)	0.828
Neutrophilic granulocytes	34%	26%	1.3 (0.7–2.4)	0.513
Haemosiderophages	16%	10%	1.6 (0.6–4.4)	0.554
Hyperaemia	64%	60%	1.1 (0.8–1.5)	0.837
Oedema	36%	52%	0.7 (0.4–1.1)	0.158
Haemorrhage	38%	32%	1.2 (0.7-2.0)	0.675
Haemorrhage accompanied by inflammatory reaction	6%	6%	-	-

### Other lesions

The prevalences of piglets with lesions in other organs are listed in Table 4. In total, 93 and 97 skin ulcerations (excluding umbilical ulcerations) were registered in Groups 1 and 2, respectively, and the average number of ulcerations per piglet was 1.9 for both groups.

**Table 4:** Prevalence of female piglets with gross lesions in the skin (i.e. ulcerations), gastrointestinal tract (i.e. gastric hyperkeratosis, intestinal haemorrhage, and/or mucosal hyperaemia), kidney (hydronephrosis, infarcts), middle ear (otitis media), and joints of front legs (synovial oedema, hyperaemia, hyperplasia, and/or haemorrhage) in Groups 1 and 2, respectively. Piglets in Group 1 were caught, lifted, and carried by one hind leg while piglets in Group 2 were caught, lifted, and carried with support under the abdomen. Skin ulcerations did not include umbilical ulcerations

Gross manifestations	Group 1 *n* = 50	Group 2 *n* = 50
Skin (ulcerations)	88%	90%
Tail	50%	60%
Carpus	40%	52%
Head	36%	26%
Other body location	22%	20%
Joints of the front legs	32%	30%
Kidney	6%	10%
Gastrointestinal tract	6%	8%
Middle ear	0%	2%

## Discussion

Female piglets caught, lifted, and carried by one hind leg did not have a statistically significant increased risk of omphalitis and umbilical hernia compared to piglets caught, lifted, and carried with support under the abdomen at 14 days of age. Lifting piglets by the hind legs is an accepted handling procedure in conventional pig production [20, 21]. However, it has been hypothesized that this procedure causes a strain on the abdominal wall thereby increasing the risk of developing omphalitis and umbilical hernia [5, 19]. In the present study, more supportive handling of piglets did not significantly reduce the prevalence of piglets with gross and most histological lesions in the umbilicus nor did it affect the thickness of the abdominal wall or distance between the abdominal wall musculatures. An exception to this was the infiltration of haemosiderophages in the umbilicus. The risk of having haemosiderophages in the umbilicus was 1.4 times higher in piglets caught, lifted, and carried by one hind leg compared to piglets caught, lifted, and carried with support under the abdomen. Haemosiderophages are haemosiderin-containing macrophages and their presence reflects an earlier haemorrhage in the tissue caused by e.g. trauma and/or infection. The prevalence of piglets with haemorrhage was equal between the groups. However, haemosiderophages have been seen in 8–35 days old porcine experimental ulcerations and human skin ulcerations of three days and older [28, 29]. Based on this, the haemosiderophages present are most likely a result of haemorrhage during the first week of life.

Umbilical hernias were present in 14% and 12% of piglets in Groups 1 and 2, respectively. The high prevalence of piglets presenting hernias is comparable to a study of 30 conventional Danish pig herds in which the prevalence of piglets with UOs ranged from 0.8 to 13.6% between herds [5]. The cause of umbilical outpouchings is suspected to be multifactorial, and risk factors include e.g. infectious omphalitis, inflammation, genetic factors, and sex [7, 10, 30, 31]. Regarding the latter, female pigs are at an increased risk of developing umbilical outpouchings compared to males [5, 10]. Based on this, only female piglets were included in the present study. A significant prevalence of pigs with umbilical hernia negatively affects productivity and animal welfare [5, 7, 8]. However, the prevalence of umbilical hernia in piglets may not reflect the prevalence in weaners and slaughter pigs as UOs may appear or undergo spontaneous regression as the pigs grow [5, 10, 32].

In both groups, some piglets were treated with antimicrobials due to diarrhoea, lameness, or nonregistered symptoms. The number of treated piglets was evenly distributed between the two groups. Antimicrobial treatments could potentially reduce the prevalence of piglets presenting omphalitis. However, there is no indication of this as 60% and 100% of the treated piglets in Groups 1 and 2, respectively, presented omphalitis.

In both groups, more than half of the piglets presented omphalitis. Given the limited study population of 100 female piglets from one herd, the results might not be representative of 2-week-old piglets in general. Unfortunately, few studies on omphalitis in piglets exist. Hovmand-Hansen et al. 2021, performed a clinical examination of 2617 piglets at two weeks of age and found 141 cases of omphalitis [10]. In addition, Searchy-Bernal et al. 1994 reported an incidence of umbilical lesions in 9 (3 by clinical examination and 6 at necropsy/slaughter) out of 2958 pigs followed from birth to slaughter [7]. The higher prevalence of omphalitis found in the present study, compared to the lower prevalence reported in other studies, may reflect that histopathological evaluation of the umbilicus identifies more cases of omphalitis than clinical examination alone. Clinically, the diagnosis of omphalitis has been based on changes such as erythema, swelling/enlargement of the umbilicus, and/or purulent discharge from the umbilical cord or periumbicular tissue in piglets and heifers [10, 33]. In the present study, none of the piglets presented with a purulent discharge at the umbilicus that was visible from the outside, i.e. before necropsy. Such cases are probably difficult to diagnose based on a clinical assessment, which might explain the remarkably high prevalence of omphalitis in our study compared to the studies with a clinical approach. Enlargement of the umbilicus is a diagnostic criterion used for diagnosing omphalitis based on clinical examination [10, 33]. In the present study, the depth, length, and width of the umbilicus were increased in piglets with omphalitis compared to piglets with no lesions (Fig. 4). However, the differences (measured in mm) seem too small to have a practical relevance.

Gross and histological lesions in the joints of the hind legs were observed in piglets from both groups. However, the prevalence of piglets presenting joint lesions did not differ significantly between the groups. This indicates that piglets caught, lifted, and carried by one hind leg did not have an increased risk of joint lesions compared to those caught, lifted, and carried with support under the abdomen. Herd personnel were instructed to catch, lift, and carry piglets in Group 1 by one hind leg, without specifying which leg to use. Consequently, piglets were not necessarily lifted by the same hind leg each time, which could influence the results. Nonetheless, by not selecting a specific hind leg, the handling procedure of piglets in Group 1 represents how piglets are typically caught and lifted in pig production.

Skin ulcerations were frequent in both groups and most often located on the tail, umbilicus, and carpus. The prevalence of pigs with skin ulceration is comparable to the prevalences reported in other studies on skin ulcerations in piglets and nursery pigs [34, 35]. Heimann et al. 2024, found front leg skin abrasion in 73% of 1045 piglets and concluded that lesions were induced by the floor [35]. Additionally, in a study of 266 pigs that died during the nursery period, 70% presented with one or more skin ulcerations [34]. Skin ulcerations are painful, at least in the acute stage, and may serve as a portal of entrance for bacterial infection. Therefore, management interventions should be implemented to prevent skin ulceration in pigs.

## Conclusion

Female piglets caught, lifted, and carried by one hind leg did not have a statistically significant increased risk of umbilical hernia, omphalitis, and joint lesions in the hind legs compared to piglets caught, lifted, and carried with support under the abdomen. The risk of haemosiderophages in the umbilicus was 1.4 times higher in piglets caught, lifted, and carried by one hind leg compared to piglets caught, lifted, and carried with support under the abdomen. This suggests that piglets lifted by one hindleg may have a higher risk of umbilical haemorrhage during the first week of life compared to those handled with abdominal support. Except for this, the method of handling did not affect gross and histological lesions in female piglets.

## Data Availability

The datasets are available from the corresponding author upon reasonable request.
